# Relating Diseases by Integrating Gene Associations and Information Flow through Protein Interaction Network

**DOI:** 10.1371/journal.pone.0110936

**Published:** 2014-10-31

**Authors:** Mehdi Bagheri Hamaneh, Yi-Kuo Yu

**Affiliations:** National Center for Biotechnology Information, National Library of Medicine, National Institutes of Health, Bethesda, MD, United States of America; Koç University, Turkey

## Abstract

Identifying similar diseases could potentially provide deeper understanding of their underlying causes, and may even hint at possible treatments. For this purpose, it is necessary to have a similarity measure that reflects the underpinning molecular interactions and biological pathways. We have thus devised a network-based measure that can partially fulfill this goal. Our method assigns weights to all proteins (and consequently their encoding genes) by using information flow from a disease to the protein interaction network and back. Similarity between two diseases is then defined as the cosine of the angle between their corresponding weight vectors. The proposed method also provides a way to suggest disease-pathway associations by using the weights assigned to the genes to perform enrichment analysis for each disease. By calculating pairwise similarities between 2534 diseases, we show that our disease similarity measure is strongly correlated with the probability of finding the diseases in the same disease family and, more importantly, sharing biological pathways. We have also compared our results to those of MimMiner, a text-mining method that assigns pairwise similarity scores to diseases. We find the results of the two methods to be complementary. It is also shown that clustering diseases based on their similarities and performing enrichment analysis for the cluster centers significantly increases the term association rate, suggesting that the cluster centers are better representatives for biological pathways than the diseases themselves. This lends support to the view that our similarity measure is a good indicator of relatedness of biological processes involved in causing the diseases. Although not needed for understanding this paper, the raw results are available for download for further study at ftp://ftp.ncbi.nlm.nih.gov/pub/qmbpmn/DiseaseRelations/.

## Introduction

Discovering disease-disease similarities could be helpful in better understanding the underlying causes of diseases and may even be useful for therapeutic purposes, as similar diseases might have similar drug targets. Disease similarities, of course, can be investigated at different levels and from different perspectives. Phenotype similarity is perhaps the most obvious way to classify diseases. This is usually the approach taken in many disease databases including Medical Subject Headings (MESH) [1] and Disease Ontology (DO) [2]. Although this method of classification is very useful, other metrics of similarity could significantly improve our understanding of the biological processes involved in similar diseases. For diseases with genetic causes, disease-disease associations could also be based on whether or not two diseases are associated with the same genes. This would extend the concept of similarity, because different phenotypes could be related to the same set of genes. However, there are similar diseases that do not share gene associations. A similarity metric that could suggest deeper relationships between diseases is therefore desirable.

Network-based similarity measures have gained popularity over the last few years. For example, Goh *et al.* [3] introduced a human disease network by treating the diseases as nodes and by linking the diseases if they had at least one shared gene association. They showed that their network was clustered according to disease classes, although they did not define a quantitative metric to find the distance between diseases in a given pair. Using a similar approach, Lee *et al.* [4] constructed a metabolic network, where nodes (diseases) were connected if mutated enzymes associated with them catalyzed adjacent metabolic reactions. They found that connected diseases had higher comorbidity than those without any link between them. Zhang *et al.* [5] constructed an extended human disease network by adding new gene associations (and so new disease links) inferred based on protein-protein interaction data. Hidalgo *et al.* [6] created a phenotypic disease network with phenotypes as nodes. The phenotypes were then linked if they had significant comorbidity. They used two different (but related) comorbidity measures based on the disease history data of a large population of patients. On the other hand, Linghu *et al.* [7] used a network in which the nodes represent genes. They integrated different functional associations, including protein-protein interactions, using a Bayes classifier whose output was then used to weight the links between the genes based on their overall functional associations. For 110 diseases, the disease genes were then prioritized according to their associations with previously known disease genes. They also calculated a measure of similarity between any two diseases based on the mutual predictability of known gene associations of one disease from the known genes related to the other disease. In another study, Suthram *et al.* [8] used mRNA expression and protein-protein interaction networks to find quantitative similarities between 54 human diseases. Mehren *et al.* [9] developed a gene-disease association database by integrating several sources and classified diseases using graph clustering algorithms. They found common functional modules for related diseases, a concept that has been reported in most network-based studies of human diseases [10]. In a recent study, Zitnik *et al.* [11] used a data mining approach to discover disease-disease associations. They introduced relation matrices describing the associations between different types of objects (genes and diseases, for example) and minimized an objective function to factorize these matrices to ones with lower dimensions, consequently clustering the diseases. Zitnik and co-workers used several types of data as constraints in their objective function including protein-protein interactions, although they concluded that these interactions were not as essential as other data in their analysis. Gulbahce *et al.* [12] created a viral disease network and introduced a local impact hypothesis stating that in this network genes associated with virally implicated diseases are located near viral targets. MimMiner, introduced by van Driel *et al.* [13], is another method to relate diseases. Unlike previous approaches, MimMiner uses text mining to assign pairwise similarity scores to more than 5000 diseases.

Although many disease-disease similarity models have been proposed, a method that uses the entire protein interaction network (not just the nearest neighbors) to define pairwise similarity is not yet in use. In this paper a simple similarity measure (called correlation) is defined between any two diseases that have gene associations. In our model a disease-protein network is created by combining disease-gene association and protein-protein interaction databases. In this network the diseases are boundary nodes; i.e. they are not connected to each other, but they are linked to the proteins (products of genes) that are associated with them. The proteins are connected based on their curated binary interactions, and the information flow in the network is modeled by a random walk starting from and ending at each disease [14], [15]. Each protein can then be assigned a weight; i.e. the expected number of visits to it. In other words, corresponding to each disease, there is a set of weights associated with the proteins (genes) in the network. From the perspective of using random walk to rank the nodes in the network, our approach is somewhat similar to that of Li and Patra [16]. On the other hand, from the viewpoint of outputting pairwise disease similarities, our method is very similar to that of MimMiner. The method of Li and Patra [16] uses a phenotype similarity network, created using MimMiner similarity scores, in addition to the gene-phenotype and protein interaction networks. Furthermore, their method was developed primarily for gene-disease association prediction. In comparison, the method presented here makes no assumptions about disease-disease similarities. We define the similarity or correlation between any two diseases based on their corresponding gene weights.

We have used our method to calculate correlations between all disease pairs present in the network. We show that higher correlations imply higher probabilities for the diseases to be from the same family of diseases and also higher likelihood of sharing biological pathways. We have compared the results of our method with those of MimMiner since both methods output pairwise disease similarities. It is shown that the results of the two methods complement each other.

We have also compared our method with those of Li and Patra [16] as well as Goh *et al.* [3] in terms of finding “hidden” disease-disease associations. Combining our method with enrichment analysis, we suggest possible disease-pathway associations and find biological pathways that might be shared between different diseases. Finally, we show that clustering diseases based on their correlations increases the number of hits found by the enrichment analysis.

## Methods

### Disease and gene-disease association databases

Curated disease and disease-gene association data were retrieved (in August 2013) from the Comparative Toxicogenomics Database (CTD) [17], North Carolina State University, Raleigh, NC and Mount Desert Island Biological Laboratory, Salisbury Cove, Maine (URL: http://ctdbase.org/). The CTD disease database merges the hierarchical MESH (Medical Subject Headings) [1] and the flat OMIM (Online Mendelian Inheritance in Man) [18] databases, where OMIM diseases are either merged to the most appropriate MESH terms or are added as children of MESH diseases [19]. The gene-disease associations reported in the CTD database are either based on direct evidences or are inferred. To reduce the uncertainty in the gene-disease associations, we ignored the inferred associations in this study. Also, only the most specific human diseases (the ones with no children) were included in the network.

### Protein-protein interaction database

To uncover how gene groups associated with different diseases are related to one another in the context of protein-protein interactions, a protein-protein interaction database is needed. We used ppiTrim [20] to create such a database. By processing iRefindex [21], which incorporates entries from all major protein interaction databases, ppiTrim can produce a protein-protein interaction database in a consistent way and without redundancies [20]. All required input files for ppiTrim were downloaded on June 6 2013, and the program was run on the same day to produce the protein-protein interaction network used in this paper.

### Disease-protein network

The disease-protein network was created by combining the CTD gene-disease association database and the protein-protein interaction network produced by ppiTrim. Naturally, only diseases associated with proteins in the ppiTrim-produced database were included in the network. An undirected graph, consisting of 16973 nodes (2548 diseases and 14425 proteins) and 214337 edges, was created by connecting the included diseases to their associated proteins, each of which is a node in the binary interaction network produced by ppiTrim. It was found that for fourteen diseases the associated proteins were disconnected from the rest of the network. These diseases were excluded in the subsequent analysis of the results (leaving 2534 diseases) because the network cannot provide more information about them.

### Information flow and disease-disease correlations

Modeling information flow by a random walk with damping, ITMProbe [14], [15] is useful in studying information flow in protein networks. Under this method, the random walk starts from one or more *source* nodes and either dissipates or ends at *sink* nodes. Source and sink nodes are also called boundary nodes, while other nodes that are neither sources nor sinks are called *transient* nodes. The ITMProbe program outputs the *expected* number of visits to each transient and sink node by random walkers originated from every source node. In this study ITMProbe was applied to the disease network described in the previous section with all diseases specified as both sources and sinks, all proteins specified as transient nodes, and with a damping factor of 0.85 (for a discussion on the effects of changing the damping factor and also the rational behind using the value 0.85 please see [15]). If we consider the flow of information starting from and ending at a given disease, we can assign a weight (proportional to the expected number of visits) to each protein (transient) node. In other words, for each disease 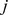, there is a corresponding vector of weights 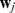 whose dimension equals the number of proteins in the network. Without loss of generality, we always normalize 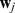 to have unit length 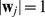 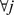.

The correlation between two diseases 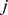 and 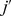 is defined by
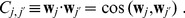
(1)The last equality results from 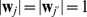. For two disconnected diseases this quantity would vanish, whereas for two diseases with the same connections to the network (diseases associated with the same set of proteins) the correlation would be unity. Disconnected diseases were not included in the analyses and so disease correlations would be positive.

For later convenience, let us also define the average correlation 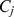 between disease 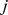 and the rest of the diseases
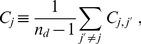
(2)where 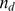 is the number of diseases under consideration. We may also define the average pairwise disease correlation 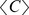 by
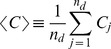
(3)In this study, we often sort pairwise correlations and bin them. Within such a bin, the average of variable 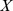 is generally denoted by 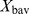, where the subscript 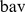 stands for bin-averaged, with
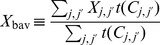
(4)where 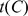 is an indicator function taking the value 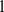 if 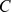 is inside the bin of interest and 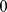 otherwise. When there is only one bin, 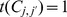 for all 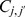 and 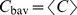 as expected.

### Enrichment analysis

In the previous section, the construction of the weight vector associated with a given disease via ITMProbe was described. Evidently, when two diseases have similar weight vectors, they are related from the perspective of protein interaction network. To provide a biological explanation of each weight vector obtained, one would need an enrichment analysis: i.e. one should find the biological terms that best describe the weight vector from an annotated term database such as GO [22] or KEGG [23]. In this study, the enrichment analysis was done using Fisher's exact test, because this is among the most used approaches.

Although there are different implementations of Fisher's exact test, we chose to use Saddlesum [24] because it is an in-house tool and it has already integrated the two term databases (GO and KEGG) of interest for the analyses. For each weight vector, the cutoff (minimum weight included in the analysis) for Fisher's exact test was chosen to be 0.001 times the mean of the ten largest weights. This variable cutoff was chosen to, in the case of more uniform vectors, prevent including too many genes in the analysis that could result in false term associations. Terms with E-values less than 0.01 were considered significant. For each disease, we used this approach to find the corresponding GO (only terms in the “biological processes” family) and KEGG terms, providing yet another way to compare different diseases.

### Clustering

To elucidate the relationships among diseases, a two-stage clustering procedure was employed. At the first stage, the diseases, each associated with a weight vector, were clustered probabilistically based on their vectors' correlations with cluster centers' vectors. An enrichment was then done for each of the final cluster centers. After the first stage, however, many cluster centers were found to be associated with similar, or even identical, sets of GO/KEGG terms. In terms of biological inferences, it was therefore necessary to do the second stage of clustering to group cluster centers based on the similarity of biological terms associated with them.

Although many algorithms are already available for clustering vectors, they usually result in non-overlapping clusters. Many diseases, however, belong to more than one family. Therefore, it is important to have a clustering method that produces overlapping disease classes. Thus, we introduced a probabilistic algorithm in which each disease was iteratively assigned a probability for belonging to a particular cluster. Each cluster was characterized by its center, a vector containing a set of weights for all proteins in the network. For a disease, the probability of being in a cluster was defined to be proportional to the cosine similarity between the weight vectors associated with the disease and the cluster center. The center vectors were initially chosen to coincide with the diseases' weight vectors. In other words, at first the number of clusters was the same as that of the diseases. The initial probabilities were computed using these initial centers. In each iteration the new positions (vectors) of the cluster centers were calculated using
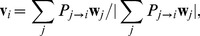
(5)where 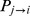 denotes the probability of disease 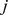 to be in cluster 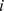, 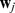 is the vector associated to the disease 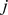 provided by ITMProbe, and the vector 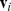 representing cluster 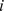 is always normalized to have unit length. The probabilities were then recomputed and used in the next iteration. Since, in this approach, the cluster centers are basically the average of the disease weights, after each iteration they would move closer to each other. For this reason before each iteration the cosine similarities between cluster centers were calculated and the centers that were closer than a cutoff were combined. Specifically, if two centers 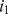 and 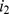, containing 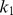 and 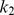 diseases respectively, had the highest cosine similarity and if their similarity was more than the cutoff, the two centers were merged to arrive at a new center vector 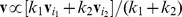 that carries with it 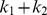 diseases. This procedure was continued until there was no pair of centers with a similarity bigger than the cutoff, which was set to 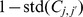 where 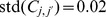 is the standard deviation of the diseases' pairwise correlations (see the Result section). The rational behind this choice is that our model cannot distinguish between two vectors if they are closer than this cutoff. It is worth noting that this process of elimination was performed even before the first iteration, because there were a number of diseases (initial cluster centers) whose correlations was more than the cutoff.

If this iterative method were continued long enough all cluster centers would be combined and there would be only one cluster. The goal of clustering was to group only highly correlated diseases, and so the iterations had to be stopped at an appropriate point. Two observations help us to find this point. First, it is desirable to express the diseases in terms of the lowest number of parameters possible, meaning a lower number of clusters. Second, a disease should be associated with fewest number of clusters possible. This is especially true for diseases that have low correlations with all other diseases (see the Results section). Such a disease should mainly belong to only one cluster (consisting only of that disease). Combining these competing observations, we stopped the iterations when the following quantity was minimized:
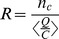
(6)where 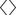 denotes averaging over all diseases, 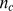 is the number of clusters, 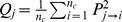, and 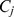 (see Eq. (2)) is the average correlation between disease 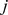 and all other diseases. For disease 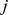, 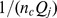 is the participation ratio [14] and 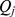 varies between 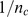 (when the disease belongs to only one cluster) and 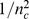 (when the disease is associated with all clusters with the same probability). Therefore, a large 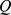 means the disease is mainly associated with only a few clusters. In the denominator of eq. (6), the contribution of diseases that are far from all others is larger due to the presence of 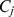. This would make it unlikely for these diseases to be in more than one cluster when the iteration is stopped. The quantity 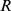 went through a minimum after 10 steps (See Fig. S1 in the first section of Supporting Information S1) at which point the iteration was stopped and the final probabilities were calculated. This procedure resulted in 1707 clusters.

Enrichment analysis was then performed for the cluster centers with the same parameters as before, except that the maximum number of genes involved was limited to 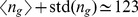 where 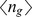 is the average number of genes included in the enrichment analysis per disease and std denotes standard deviation. This limit was imposed because averaging weight vectors results in a vector of more uniformly distributed weights and so the number of included genes could reach much higher values than before. The E-values corresponding to the terms found by the enrichment analysis could be used to create a new vector 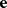 for each disease or cluster center with term association. For this purpose the union of all significant terms associated with all centers was determined. For cluster 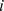 the 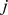
th component of 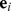 was then defined as 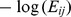 if 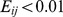 and zero otherwise. Here 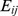 is the E-value corresponding to the 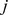
th term when querying SaddleSum using 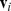. The 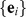 vectors were then used for the second stage clustering.

In the second stage, the clusters obtained in the first stage were separated into two groups depending on whether or not they had been associated with biological terms. The first (with term associations) and second group had 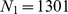 and 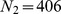 members respectively. For the first group, the set of vectors 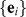 were then defined and were clustered using a distance-based hierarchical approach. The distance between 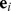 and 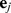 was defined as 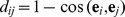, and the cutoff was chosen to be 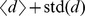. To avoid over-clustering, we used a more stringent similarity measure here than the one used before for comparing the terms assigned to different diseases. For disease 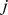 the probability of being in the 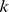
th new cluster, containing 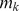 clusters from the first group, was defined as 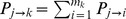 where 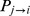 is the probability of disease 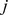 belonging to the 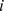
th cluster in the first group after the first stage of clustering.

We also used Cfinder [25], [26], a program for clustering nodes of a graph, to create overlapping clusters of diseases. The advantage of Cfinde over similar algorithms is that, like the clustering method explained here, it produces overlapping clusters. To use Cfinder, we first created a disease network by connecting each pair of diseases with an edge weighted by their correlation. Using this method, we obtained similar results to those from our clustering algorithm described above. For a detailed description of the procedure and the results please see Supporting Information S1.

### Evaluating the accuracy of p-values

The accuracy of p-values reported (by Saddlesum) for the original (not averaged) weight vectors has already been evaluated by Stojmirovic and Yu [24]. The cluster centers, however, are weighted averages of all weight vectors. To investigate whether or not averaging affects the accuracy of the p-values (and consequently the E-values) reported by the enrichment analysis, we took a similar approach to that of Stojmirovic and Yu [24] to calculate the “empirical” p-values and to compare them to the reported ones. Briefly, the gene list was shuffled 672 times and, for each gene list, enrichment analysis was performed for all cluster centers and the reported p-values were recorded. This number (672) was chosen to have approximately 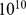 weight-term matches, i.e. 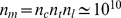, where 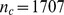 is the number of clusters, 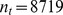 is the total number of GO/KEGG terms, and 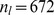 is the number of randomized gene lists. The empirical p-value corresponding to the cutoff value 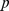 was then defined as 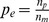, where 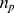 is the total number of reported p-values (for all cluster centers and all gene lists) that are smaller than or equal to 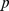. The results, given in Fig. S2 and section 2 of Supporting Information S1, showed that the reported p-values were indeed accurate.

## Results

### Statistics of disease-disease correlations

Based on the proteins that the two diseases were connected to, disease pairs were classified into three categories. If both diseases in a pair were connected to the same set of proteins, the pair was assigned to category (1). Members of this category, by definition, had the largest correlation possible (unity) and were equivalent in our study. The number of “independent” (not equivalent to any other) diseases was 1962. If, on the other hand, the two diseases shared some (but not all) associated proteins, the pair was classified in category (2). Category (3) consisted of the rest of the disease pairs (the ones with no shared connections to the protein network). Category (1), (2), and (3) had 978, 5243, and 3203090 pairs respectively.

Using eq. (1), the 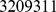 (total number of pairs from all three categories) pairwise correlations 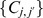 were calculated. The median, mean and standard deviation of 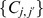 were 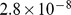, 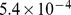, and 0.02 respectively. These statistics indicate that although the correlations were generally very small, there was a large number of outliers (disease pairs with high correlations compared to the mean). Obviously, disease pairs in category (1) had the largest correlation possible (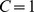). The members of the second category had overall larger correlations (with a mean value of 0.13, a standard deviation of 0.23, and a median of 0.03). However, some pairs in this class had low correlations (with a minimum of 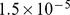). In fact 179 disease pairs in the third category (pairs with no common gene associations) had higher correlations than the median correlation of pairs in category (2), indicating that having some shared network connections does not necessarily translate to high correlations, although 95.9% of disease pairs in the second category had larger than average correlations. The maximum correlation of the third category, containing 99.8% of the disease pairs, was 0.237.

Interestingly, the average correlation between a disease and all others varies dramatically. In fact the lowest average correlation (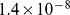) was more than five orders of magnitude smaller than the largest, suggesting that some diseases have relatively low correlations with all other diseases. For example, 286 diseases have correlations less than 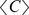 with all other diseases.

### Interpretations of correlations

To investigate what high correlation between two diseases could imply, a number of quantities were calculated. First, we calculated, for disease pairs with correlations in a certain interval, the probability of being siblings (having the same parents in the CTD/MESH database). To achieve this, the disease pairs were sorted according their correlations and were divided into bins. The probability 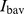 (see eq. (4)) of finding a sibling pair in each bin was then defined as the ratio of the number of sibling pairs to the total number of disease pairs in that bin. The number of disease pairs in each bin was 1000.

Second, for each disease pair, we calculated the similarity 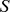 between the associated enriched terms, which was defined as the ratio of the number of significant terms shared between the two diseases to the total number of identified significant terms. In other words, for two diseases associated with exactly the same GO and KEGG terms 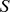 would be unity, whereas for diseases with no common terms it would vanish. If one or both diseases in a pair did not have any term associated with them, 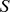 was not defined. To find the distribution of disease pairs with 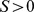, the pairs were partitioned into bins as described in the previous paragraph. In each bin, the average probability for two diseases in a pair to hit the same GO/KEGG terms was then defined as 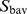 (see eq. (4)).

As expected, the enrichment analysis did not find any significant biological terms associated with some of the diseases, implying that, for some disease pairs, calculation of 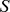 was not possible. In this study Saddlesum was able to find significant GO/KEGG terms for about 60% of the diseases (1530 out of 2534). For diseases with significant term hits, the average numbers of identified GO and KEGG terms were 34.7 and 5.2, and the standard deviations were 52.2 and 8.2. The large spread of the number of hits was due to the fact that some diseases, with a large number (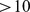) of connections to the network, had hundreds of GO and tens of KEGG terms associated with them. Overall 3182/203 unique GO/KEGG terms were hit by the enrichment analysis.

**Figure 1:**
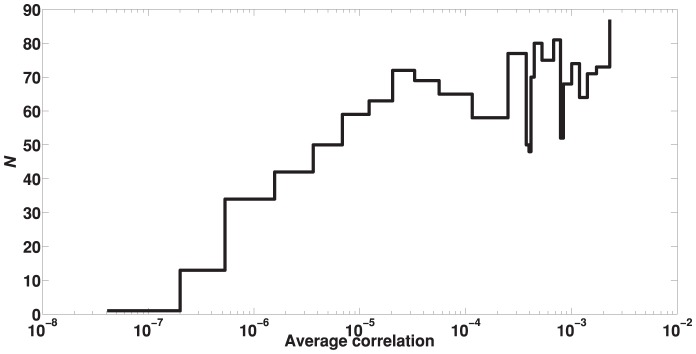
The relation between the results of enrichment analysis and the average correlation 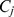. The percentage of diseases for which GO/KEGG terms were identified by Saddlesum as a function of average correlation 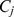. To facilitate the calculation, we sorted all 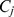
s in ascending order and placed them into bins each containing 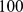 diseases. The percentage is then measured by the number 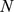 of diseases with GO/KEGG term hit(s) per bin. For very low average correlations 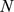 is significantly lower.

Interestingly, there was a significant difference between the percentage of pairs with undefined 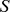 when disease pairs with low and high correlations are considered. For example, 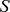 was undefined for 23% of disease pairs with correlations greater than 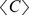, as opposed to 64% for pairs with correlations smaller than 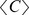. This can be understood through the fact that the percentage (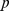) of diseases that had been successfully assigned one or more GO/KEGG terms by Saddlesum was smaller for diseases with very low average correlations. This behavior is shown in Fig. 1. After sorting 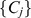 into ascending order and placing them in bins each containing 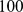 diseases, we computed the average 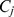 in a bin and, in the same bin, the number of diseases 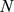 that had one or more GO/KEGG term hits. In Fig. 1, 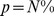 is plotted versus the average 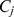 per bin and the aforementioned behavior is clearly displayed.

**Figure 2:**
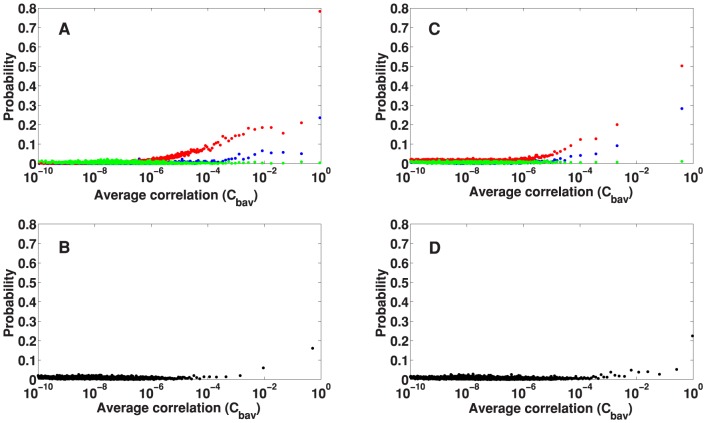
The probabilities of having common term associations or being siblings. (A) The probabilities of finding a pair of diseases with (1) common GO/KEGG terms (red), (2) the same parents and common associations (blue), and (3) the same parents without shared biological terms (green) are shown. Here only pairs with a defined term similarity are considered. (B) For pairs with undefined 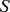 (pairs with at least one member not associated with any biological terms), the distribution of siblings is plotted as a function of correlation. (C) and (D) show similar quantities to (A) and (B) respectively, when the biological term associations are directly retrieved from the KEGG DISEASE database.

Since 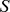 was undefined for a large number of disease pairs, the pairs were divided into two sets: with defined (first set) and undefined (second set) term similarities. For the first set (with defined 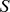), Fig. 2 (A) illustrates the behavior of 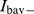
(in green), 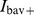
(in blue), and 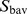 (in red), where 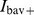 (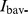) is 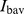 for pairs with 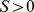 (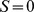). The figure clearly shows, when 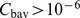, a rise in the probability of a disease pair to have common biological associations as correlation increases. The figure also indicates, when 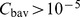, that disease pairs with higher correlations are more likely to be siblings if they have 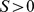. However, the siblings without shared terms have almost a flat (correlation-independent) distribution, although the percentage of such pairs is very small (about 0.5%). One possible explanation for these results is that the increase in the percentage of siblings in highly-correlated diseases is in fact due to an increase in the percentage of the pairs with 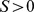. In other words, in high correlation regime, most of the siblings are a subset of disease pairs with shared GO/KEGG terms. Figure 2 (B) shows how 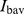 varies with correlation for the second set of disease pairs (the ones with undefined term similarities).

Using only the terms from the *manually* curated disease-pathway associations [23] in the KEGG DISEASE database (downloaded on December, 12 2013) in place of the terms retrieved from enrichment analyses, term similarities (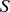) and the probabilities 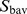, 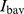, 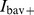, and 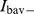 were recalculated. In this KEGG database, one or more OMIM diseases are associated with one or more KEGG pathways. The CTD disease database [17] was used to find the equivalent MESH diseases that were used in our study. The results are shown in Figs. 2 (C) and (D). Overall, the trends observed in these figures are very similar to those shown in 2 (A) and (B): i.e. increase in 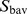/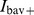 with correlation (for 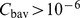) and correlation-independence in 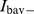 for pairs with defined 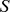, and a uniform and then increasing 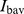 for pairs with undefined term similarities.

We have also computed the degree of overlap between the enrichment analysis and KEGG DISEASE database. For each disease 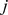, out of the total number of annotated KEGG pathway associations 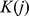 we calculated the number of associations 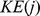 that were also reported significant by enrichment analysis. The ratio 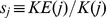, for disease 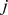, measures the agreement between the curated pathway assignment and the enrichment analysis. There were 490 diseases that had been annotated in the KEGG DISEASE database and also had term hits using the enrichment analysis. The average value of 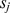 for these 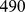 diseases was found to be 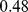, indicating on average 48% of the annotated terms for each disease in the KEGG DISEASE database were also found using our enrichment analysis.

### Comparison with MimMiner and human disease network

MimMiner [13] uses a text mining approach to calculate a pairwise disease similarity score that, like correlation defined here, ranges from 0 to 1. However, since these two measures have very different distributions and are defined based on different concepts, they cannot be directly compared. For this reason we adopted the procedure described in the third section and Fig. S3 of Supporting Information S1 to find equivalent cutoff correlations and MimMiner scores, 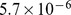 and 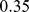 respectively, and to compare the two methods.

In the absence of a true gold standard, we used disease-pathway associations reported by KEEG DISEASE databse [23] to compare the retrieval agreement with KEGG DISEASE from MimMiner and that from our model. This was done by comparing the effectiveness of the two methods to identify disease pairs that are associated with the same biological pathways as annotated in KEGG DISEASE. To make a fair comparison, only disease pairs with defined term similarities 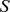 (see the “Interpretations of correlations” section) and with available MimMiner scores were included in this analysis (336610 pairs, about 10% of all pairs). By ranking the disease pairs based on either their correlations or their MimMiner scores, two lists of pairs were created. For each list, the weighted number of disease pairs with common associated biological terms that were among the first (highest ranking) 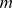 pairs was calculated as 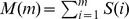, where 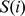 is the term similarity between diseases of pair 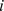 using KEGG DISEASE as the standard. The function 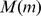 provides a measure for comparing the two methods: a faster rise in 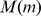 would mean a larger number of pairs with high term similarity have been ranked higher than the others, indicating a better agreement with KEGG DISEASE.

**Figure 3:**
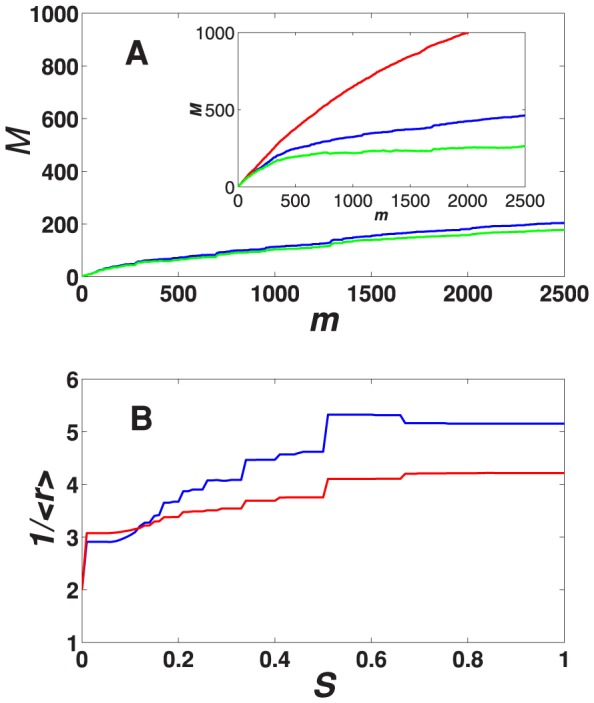
Comparison with MimMiner. (A) The inset figure shows the number (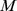) of weighted disease pairs with shared KEGG pathways that were ranked higher than 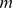 by MimMiner (in red) and or by our method (in blue). Also shown in the inset (in green) is the weighted number of pairs with common term associations missed (ranked lower) by MimMiner, but identified (ranked higher) by our model. In the main panel, the same quantities corresponding to the proposed method are plotted after exclusion of obvious candidates for being related. The closeness between the blue and green curves indicates that the non-apparent candidates found by our method are largely missed by MimMiner. Displayed in panel (B) is the inverse of average normalized rank versus the term similarity cutoff. At large similarity cutoff, the higher the average normalized rank (the smaller 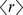 and thus the larger 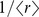) the better the agreement between the quality scores (cosine similarity or the MimMiner score) and the KEGG annotation.

The results (MimMiner in red, our method in blue) are shown in the inset of Fig. 3 (A). The green curve shows the weighted number 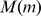 of disease pairs identified (ranked higher than 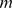) by our method, but missed (ranked lower) by MimMiner. Similar trends are observed for both methods, but a better performance (faster rise in 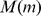) for MimMiner is indicated. This finding is expected, because MimMiner is based on mining the literature, which is also the source of the *manually* curated data in the KEGG DISEASE database. However, an important observation is that the two methods do not find the same pairs, especially in terms of less apparent relationships. To see this feature, we first excluded the disease pairs that were obvious candidates for being related, i.e. sibling diseases and pairs with common gene associations (3847 pairs were excluded leaving 332763). We then recomputed the blue and the green curves, shown in Fig. 3 (A). The closeness between these two curves indicates that for non-apparent relationships, the disease pairs identified by our method are largely missed by MimMiner. In Fig. 3 (A), about 87% of pairs ranked higher than 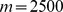 (equivalent to a correlation of 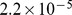 and a MimMiner score of 0.41) by the method presented here were missed by MimMiner.

Given the fact that our method and MimMiner effectively find different pairs, one may wish to look at the quality of retrieval using a different measure other than 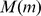. In Supporting Information S1, we have described how to find the cutoff cosine similarity and MimMiner score, above which there exists an apparent positive correlation between the similarity/score and the pair relatedness. Denote the number of disease pairs with similarity/score above the cutoff by 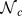/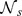. We defined the normalized rank 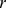 as rank divided by 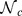 or by 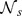 depending on whether cosine similarity or MimMiner score was in use. For a given cutoff term similarity 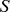, we first found among 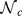 or 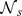 disease pairs with term similarity larger than 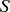, and then computed their average normalized rank 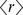 when all pairs were ranked either by correlation (cosine similarity) or by MimMiner score. Evidently, for large cutoff 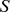, a larger 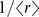 indicates a better retrieval fidelity. Using this measure, results shown in Fig. 3 (B), our methods seems to provide higher retrieval fidelity. One should bear in mind, however, comparisons of such should always be taken with a grain of salt due to the difficulty in constructing a gold standard and totally impartial datasets.

It is also worth noting that for a very large subset (90%) of disease pairs investigated in this study, KEGG term similarities or MimMiner scores were not defined. However, many of these disease pairs, or the ones with 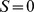 or with a MimMiner score of zero, may in fact be related. For example, 5090 (97%) of disease pairs in the second category and 838 (86%) of the members of the first category of pairs, which have common gene associations and are more likely to be related, were in this subset. Even diseases with no gene associations or the ones that have been classified in totally different families could share biological pathways or phenotypic similarity. On the one hand, many pairs with documented relationships may not have yet been annotated by KEGG DISEASE or scored by MimMiner, or may have been reported as being not related. Table 1 lists ten example pairs, with correlations much larger than 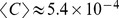, from all three categories of disease pairs. On the other hand, it is likely that some disease-disease relationships have not yet been discovered. One should keep in mind that the members of the majority of pairs with significant correlations in our study have no obvious relationships (do not share genes and are not siblings), and also their possible relationships have not yet been experimentally verified. From a practical point of view, however, these pairs are more interesting, because they suggest unknown and non-trivial relationships that, if verified, could add to our knowledge about the causes and possibly cures of certain diseases.

**Table 1:** Examples of relationships between diseases that have undefined (or zero) term similarity and undefined (or zero) MimMiner score, and are from different disease families.

First disease ID	Second disease ID	First disease name	Second disease name	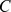	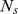	Relationship
MESH:C567070	MESH:C536289	Atypical mycobacteriosis, familial, X-Linked 1	Immunodeficiency without anhidrotic ectodermal dysplasia	1.0	1	Both diseases have been associated with nuclear factor kappa B signaling [31], [32].
MESH:C536198	MESH:C536113	Ehlers-Danlos syndrome type 6	Nevo syndrome	1.0	1	These diseases have been suggested to be identical [33].
MESH:C537494	MESH:C566453	Stickler syndrome, type 3	Deafness, autosomal recessive 53	1.0	1	Hearing loss is one of the symptoms of Stickler syndrome, type 3 [34].
MESH:C535407	MESH:D053609	Gamma aminobutyric acid transaminase deficiency	Lethargy	0.9961	1	Lethargy has been reported in pateints with Gamma aminobutyric acid transaminase deficiency [35].
MESH:D016301	MESH:C562440	Alveolar bone loss	Hypophosphatasia, childhood	0.9584	1	These are both tooth/bone diseases.
MESH:C564629	MESH:C538150	Deafness, autosomal recessive 31	Syndactyly Cenani-Lenz type	0.1040	0	Hearing loss has been associated with Cenani-Lenz type of syndactyly [36].
MESH:C536156	MESH:C536601	Keratomalacia	Amaurosis congenita of Leber, type 2	0.0835	0	These are both eye diseases.
MESH:C563906	MESH:C563425	Cardiomyopathy, dilated, 1o	Diabetes mellitus, permanent neonatal	0.0197	0	ATP-sensitive potassium channels have been reported to be involved in both diseases [37], [38].
MESH:C564334	MESH:D008527	Acrocapitofemoral dysplasia	Medulloblastoma	0.0168	0	These disease have been associated with Hedgehog signaling pathway [39], [40].
MESH:C565334	OMIM:188890	Epilepsy, nocturnal frontal lobe, type 3	Tobacco addiction, susceptibility to	0.0155	0	Both diseases have been associated with mutations in nicotinic acetylcholine receptors [41].

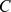
 and 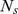 denote correlation and the number of common gene associations respectively.

From the perspective of finding “related” disease pairs with zero MimMiner score, Li and Patra [16] found 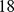 non-apparent related pairs while combining a phenotype similarity network (created using MimMiner similarity scores), a gene-phenotype network and a protein interaction network. For 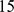 out of the 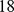 pairs, support information for relatedness was provided by Li and Patra [16]. The relatedness evidence for 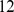 of the 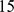 pairs is founded on that the member diseases are classified in the same disease class. Using our method, we have found 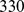 disease pairs with zero MimMiner scores, each of which has its member diseases classified under the same disease family according to MESH. We have also found 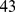 disease pairs with zero MimMiner scores, each of which has its member diseases share at least one biological pathway according to KEGG DISEASE database.

The proposed method was also compared to the Human Disease Network (HDN), introduced in the pioneering work of Goh *et al.* [3]. In this method the disease network is created by linking diseases that have common gene associations. The method proposed here, however, links the diseases based on their correlations, i.e. the diseases are linked if they have significant correlations (larger than the cutoff 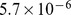, as obtained in Supporting Information S1). Interestingly, in our study the minimum correlation between diseases with shared gene associations was 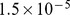. In other words, the links of a disease network created by the method of Goh *et al.* would be a small subset of those of our disease network. To find out if the additional disease-disease relations suggested by the proposed method are supported by the available experimental data, once again KEGG DISEASE database was used. We considered only diseases annotated by KEGG (1272 out of 2534 included diseases) and created three disease networks by linking the diseases using three different connectivity measures, i.e. having shared gene associations, high correlation, and having common pathway associations as annotated by KEGG DISEASE. The total number of links between the diseases in the three networks were 527, 14202, and 45577 respectively. The number of coinciding links between the KEGG-based and the correlation-based networks was 2988, as opposed to 389 when comparing KEGG and HDN networks. In other words, 2599 pairwise disease relations predicted by our method and missed by HDN are supported by the KEGG DISEASE database. Both methods however failed to predict the relationships between a large number of diseases that, according to KEGG DISEASE, have shared biological pathways. On the other hand there are many diseases that have high correlations, but are not reported by KEGG as having common pathway associations. As discussed before, these are not necessarily false positives. The KEGG database does not yet contain many literature-supported relationships (see Table 1 for some examples), but more importantly, there might be many disease relations that have not yet been discovered. An important aspect of the proposed method is that it suggests disease relationships that should be experimentally verified.

### Effect of clustering

Based on the hypothesis that highly correlated diseases are more likely to have common pathways, one can use correlation-based clustering to increase the number of hits when searching for biological terms associated with the diseases. Assuming that all diseases in a given cluster share some pathways/processes, one can increase the chance of finding these pathways/processes by weighted averaging of the weight vectors assigned by ITMProbe to the diseases in the cluster. The rational behind this method is that each vector may be contaminated with “noise” and that the “signal” could be amplified by averaging. To accommodate the scenario that a disease might belong to several families, we used a probabilistic clustering method (see Methods) that allowed overlapping clusters and assigned a probability to each disease for being in a particular cluster.

**Figure 4:**
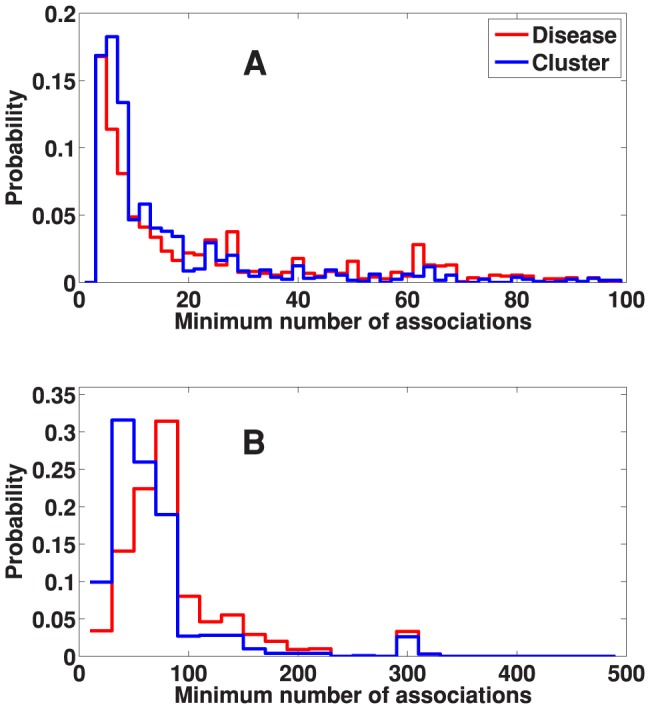
The effect of clustering on the minimum term size. The minimum term size distribution of (A) GO and (B) KEGG terms reported by SaddleSum enrichment analyses when using disease weight vectors directly (red curves) and when using cluster center vectors (blue curves). Not only the most informative (smallest size) terms are preserved during clustering, the clustering procedure seems to shift the minimum term size distribution towards the small end, indicating the likelihood of providing even more specific terms when weight vectors are grouped under the proposed clustering procedure.

Our iterative approach resulted in 1707 clusters. Enrichment analysis was run for all cluster centers obtained in this stage and found significant hits for 1301 clusters with an average of 70.9/7.5 GO/KEGG terms per cluster, which was higher than the average number of terms found for the diseases. The probabilities of belonging to different clusters were calculated for each disease and were used to determine the percentage of diseases with term hits, defined by
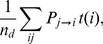
(7)with 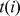 being an indicator function taking value 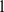 when cluster 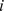 has a term hit and 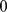 otherwise. Interestingly, the number of such diseases showed an increase from 60% (when enrichment was directly performed for the diseases) to 85%. For the diseases that had term hits using both methods (direct and through clustering) the term similarity 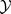, was calculated using
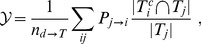
(8)with 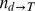 being the number of diseases that have significant term hits, 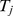 being the set of terms associated with the 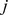
th disease, 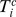 being the set of those assigned to the cluster 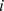, and 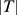 denoting the number of members in the set 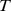. We found 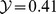. This seems to indicate that more than 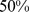 of the terms associated with the diseases were dropped upon merging to clusters and some information might have been lost in the process. What is really important, however, is whether terms of small number of annotated genes are preserved, as these terms are most specific and usually most informative. Upon examining the distribution of minimum GO/KEGG term size (number of annotated genes for that term) when running SaddleSum using diseases directly and using cluster centers, we find that the most informative terms are largely kept in the process. The distribution of the minimum term size is shown in Fig. 4.

To illustrate how clustering through weight vectors may increase the likelihood of associating a disease with terms, we examine the late-onset Parkinson's disease (OMIM:168600). This disease was not associated with any terms when enrichment analysis was directly performed for the disease. After clustering, however, the top four clusters, ranked by their probabilities of including the Parkinson's disease, were associated with the Parkinson's disease pathway. Specifically, the term hits (with E-values smaller than 1e-4) for the cluster with the highest probability (13%) are listed in Table 2, which include Parkinson's disease, Alzheimer's disease and other neurological processes.

**Table 2:** Terms associated with the cluster with the highest probability to include the Parkinson's disease.

Term ID	Name	E-value
GO:0007268	synaptic transmission	4.22e-12
GO:0019226	transmission of nerve impulse	4.58e-12
GO:0035637	multicellular organismal signaling	2.00e-11
GO:0007267	cell-cell signaling	1.13e-10
GO:0050877	neurological system process	4.54e-10
GO:0001963	synaptic transmission, dopaminergic	5.34e-08
GO:0007270	neuron-neuron synaptic transmission	4.47e-07
GO:0044708	single-organism behavior	8.73e-07
GO:0003008	system process	1.16e-06
GO:0030534	adult behavior	1.69e-06
GO:0001505	regulation of neurotransmitter levels	3.81e-06
GO:0006805	xenobiotic metabolic process	4.11e-06
GO:0071466	cellular response to xenobiotic stimulus	4.59e-06
GO:0009410	response to xenobiotic stimulus	4.59e-06
GO:0044281	small molecule metabolic process	1.62e-05
GO:0007610	behavior	5.39e-05
GO:1901615	organic hydroxy compound metabolic proce	6.38e-05
GO:0023052	signaling	6.72e-05
GO:0044700	single organism signaling	6.72e-05
GO:0065008	regulation of biological quality	7.75e-05
KEGG:hsa04080	Neuroactive ligand-receptor interaction	2.61e-19
KEGG:hsa05010	Alzheimer's disease	2.73e-06
KEGG:hsa05012	Parkinson's disease	8.69e-06

**Figure 5:**
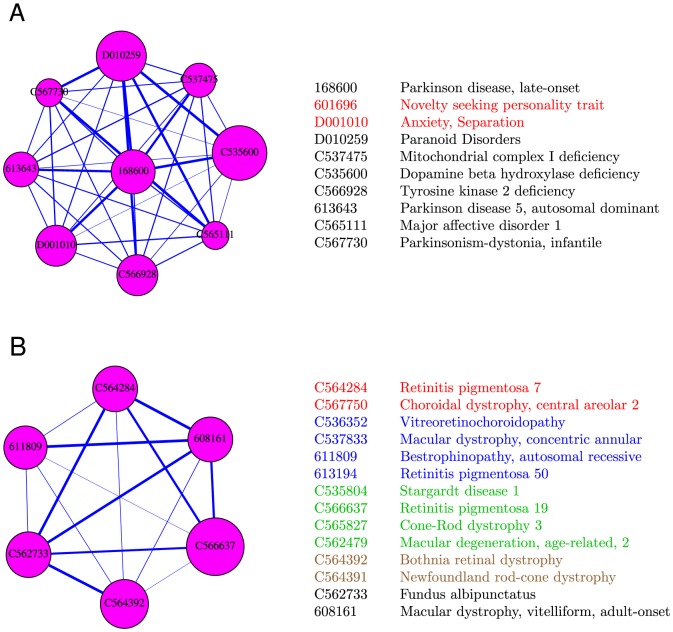
Two example clusters. The clusters that include Parkinson's disease (OMIM:168600) and Retinitis pigmentosa 7 (MESH:C564284) are shown in panels (A) and (B) respectively. In each case, only diseases with membership probabilities larger than 5% are shown. The size of each node (circle) is proportional to the probability of membership of that node in the cluster. For a disease pair, the thickness of the line linking the diseases is proportional to 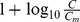, where 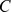 is the correlation between the two diseases and 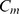 is the minimum correlation between all diseases shown in each cluster. The names and IDs of the members of each cluster are also given. Diseases whose names are written in the same color (other than black) have exactly the same gene associations and so are equivalent in our study. Equivalent diseases are represented by one node in the figure. For example, the node identified by C566637 in panel (B) represents the four diseases whose names are in green, i.e. C535804, C566637, C565827, and C562479.

Retinitis Pigmentosa is an eye disease, with many different types, which is characterized by progressive retinal degeneration. As a second example, we examined the cluster and term associations for type 7 of this disease (MESH:C564284), which had no term hit before clustering. The disease was in multiple clusters (with relatively high probabilities 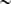
10%) that were associated with the phototransduction pathway. Given in Table 3 are the terms associated with the cluster with the highest probability (10%), which are related to phototransduction, detection of light and response to light. The phototransduction pathway, along with the Retinal metabolism (KEGG:hsa00830) and Spliceosome (hsa03040) pathways, has been indeed annotated to be related to this disease by the KEGG DISEASE database. Figure 5 visualizes the clusters that contain Parkinson's disease and Retinitis Pigmentosa 7.

**Table 3:** Terms associated with the cluster with the highest probability to include the Retinitis Pigmentosa type 7.

Term ID	Name	E-value
GO:0007603	phototransduction, visible light	5.64e-09
GO:0009584	detection of visible light	9.95e-09
GO:0007602	phototransduction	1.69e-08
GO:0009583	detection of light stimulus	2.51e-08
GO:0009582	detection of abiotic stimulus	1.12e-07
GO:0009581	detection of external stimulus	2.98e-07
GO:0051606	detection of stimulus	3.66e-06
GO:0022400	regulation of rhodopsin mediated signali	1.07e-05
GO:0016056	rhodopsin mediated signaling pathway	1.31e-05
GO:0009416	response to light stimulus	1.47e-05
GO:0009314	response to radiation	1.45e-04
GO:0071482	cellular response to light stimulus	9.23e-04
GO:0008277	regulation of G-protein coupled receptor	5.23e-03
GO:0071478	cellular response to radiation	5.23e-03
KEGG:hsa04744	Phototransduction	8.51e-04

As a third example, the associations of the Knobloch syndrome (MESH:C537209) were also investigated. This is another eye disease that is characterized by different abnormalities, including cataracts, dislocated lenses, vitreoretinal degeneration, and retinal detachment [27]. Unlike the other two examples, this disease is primarily a member of one cluster with a probability of 62%, and the second highest probability was much smaller (3%). According to the KEGG DISEASE database, the pathways involved in this disease are focal adhesion (KEGG:hsa04510), ECM-receptor interaction (KEGG:hsa04512), and cell adhesion molecules (KEGG:hsa04514). Focal adhesion, and ECM-receptor interaction were in fact among the KEGG terms that were found to be associated with the cluster. Due to the relatively large number of associated terms with this cluster, the whole list is not given.

The first two examples indicate that there was a high degree of overlap between the GO/KEGG terms associated with different clusters. In other words, several clusters have phototransduction or Parkinson's disease pathways associated with them. For this reason a second round of clustering was performed, which was based on term similarity rather than disease correlations through weight vectors, and the probabilities of belonging to each new cluster was calculated. The second stage clustering reduced the number of clusters with term associations to 217, which substantially reduced the term overlap. For example, the highest new membership probability (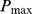) for Parkinson's disease became 31%, and the new cluster, as expected, was associated with the Parkinson's disease pathway. Similarly, after the second stage clustering, Retinitis Pigmentosa type 7 was primarily in one cluster with the probability of 44% (which is associated with phototransduction) and all other probabilities were less than 15%. On other hand, 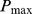 for the Knobloch syndrome only increased modestly to 73%. The large reduction of the number of clusters is consistent with the view that many diseases share common modules or biological pathways.

## Discussion

Disease networks can provide valuable information when investigating if and how two given diseases are related. In this paper a simple network-based measure, referred to as correlation, is introduced to explore possible relations between any two genetic diseases. The correlation between two diseases is defined (eq. (1)) by the inner product of their corresponding weight vectors. The weight vector associated with a disease is based on the flow of information from that disease back to itself in a disease-protein network created by integrating the available disease-gene associations and protein-protein interactions. The results obtained are therefore reflective of the data available. Although specific cases might be sensitive to the data employed, the general trend obtained should remain robust.

Our results suggest that diseases with higher correlations are more likely to be phenotypically related (be children of the same parent), and to share biological pathways as determined by enrichment analysis. Specifically, our result shows that most siblings with high correlations share at least some GO/KEGG terms. In fact, siblings with large mutual correlations are mostly a subset of diseases that have been assigned common biological terms. We also find that when enrichment analysis does not return a shared pathway (when 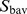 is very small), only less than 1% of disease pairs are siblings. However, correlation between diseases seems to be more an indicator of similarity of the involved biological processes than an absolute measure of phenotypic overlap. This is evidenced by a small but steady presence of sibling disease pairs with extremely low correlations. This is consistent with the view that high correlation indicates shared pathways and thus likely shared pathophenotypes, while pathophenotypic similarities might not require high correlations.

Different genes/processes may cause the same or closely related phenotypes when they effectively influence the same pathway. The clustering procedure used in this paper is aimed to find this type of event and group them together. When diseases are caused by different genes that are parts of the same pathway, the likelihood for their weight vectors to resemble one another is apparently higher than when they do not share the same pathway. Suitably averaging those weight vectors (eq. (5)) leads to a cluster center that may better represent the pathway. This procedure also has the effect of reducing the “noise” and enhancing the “signal” of the weight vectors, which is evidenced by the increase, from 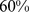 to 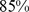, of the percentage of diseases having significant term hits upon first stage clustering.

On the other hand, since our data sources only include the disease-gene associations and the protein interactions, the regulatory effects were not included explicitly. This presents a limitation as well as points the direction for future improvement. For example, from a gene-centric point of view, being a part of one or multiple pathways, a gene could result in multiple diseases either through different states (overactive vs. underactive) in a single pathway or through influencing multiple pathways. Largely absent from the disease-protein network currently used, these subtle effects do exist. Table 4 shows a set of five diseases with shared connections to the network through low density lipoprtein receptor-related protein 5 (*LRP5*, OMIM:603506). As far as the disease-protein network is concerned these diseases are equivalent and have perfect correlations. That is, these five diseases are naturally grouped together under our method even though their annotations do not suggest such a grouping. One of the diseases (MESH:C566619) is a member of the family of eye diseases characterized by incomplete development of the retinal vasculature, and the others are musculoskeletal diseases associated with high (MESH:C536527, MESH:C536748, and MESH:C536056) and low (MESH:C536063) bone densities (MESH:C536056 and MESH:C536748 are sibling diseases). Interestingly, osteoporosis-pseudoglioma syndrome is a disease that is characterized by both low bone density and eye abnormalities. The top (with lowest E-value) GO/KEGG term assigned to these diseases by enrichment analysis was “Wnt signaling pathway”, i.e. GO:0016055/KEGG:hsa04310 (obviously, all diseases in this set had the same terms associated with them, because they share the same connection to the network). This pathway, through mutations in *LRP5*, has been indeed reported to be involved in development of diseases related to both bone density and also some eye abnormalities [28]–[30]. In these studies MESH:C536527, MESH:C536748, and MESH:C536056 have been associated with an increase in Wnt signaling, whereas underactive Wnt signaling has been reported to cause MESH:C536063 and MESH:C566619.

**Table 4:** An example of a set of diseases that are associated with the same gene, but some have different phenotypes.

Disease ID	Disease annotation	Disease family
MESH:C566619	Exudative Vitreoretinopathy 4	Eye diseases
MESH:C536527	Van Buchem disease type 2	Musculoskeletal diseases
MESH:C536063	Osteoporosis-pseudoglioma syndrome	Musculoskeletal diseases
MESH:C536748	Worth syndrome	Musculoskeletal disease
MESH:C536056	Osteopetrosis autosomal dominant type 1	Musculoskeletal diseases

Although MimMiner [13] uses a totally different approach to measure disease-disease similarity, like our method, it provides pairwise scores for a large number of diseases. Therefore, it is perhaps the most suitable method to be compared with the approach presented in this paper. A comparison between the performances of the two methods in identifying disease pairs with shared associated KEGG pathways indicated that the results of the two approaches are largely complementary. In other words, each method can provide valuable information about relationships between diseases that cannot be obtained from the other. It should be noted, however, that the KEGG DISEASE database, which was assumed as a gold standard for making such comparison, is underdeveloped and manually curated. A more complete database that does not bias towards either text-mining or gene-disease associations is needed for a sound comparison among methods outputting pairwise disease similarities.

Although using protein interaction data in conjunction with finding disease relations is not new, utilizing the information flow to find for each disease its corresponding ITM (information transduction modules) in the context of protein-protein interaction is novel. There are a number of directions that we can potentially look into but did not do so because of the lack of a comprehensive gold standard to assess them. For example, the clustering procedure proposed can be turned into a tool to classify diseases based on the underlying protein interactions. Also, it would be interesting to examine clusters without any term hits but containing multiple diseases. This might help in finding the common cause among seemingly unrelated diseases. In addition, it can be valuable to examine clusters with significant term hits but whose member diseases do not yet have annotated cause. The term hits in this case may shed some light in searching for the underlying cause of the disease. Even though we did not pursue further analyses along those directions, we have, however, compiled the clustering results and make them available for download. If properly used, these compiled results form a database for finding candidates of not-yet-solved problems in disease cause and mutual relations.

Another interesting finding of the study was the higher rate of failure of the enrichment analysis to find significant GO/KEGG terms associated with diseases that had very low average correlations with the others. This is perhaps due to the incompleteness of the network, i.e. missing protein-protein interactions or gene-disease associations. Such missing nodes would prevent both ITMProbe from finding correlated diseases and Saddlesum from assigning biological terms. Improvement in the databases used in this study to create the disease network could change the results for diseases with missing connections. However, such improvements seem to be less likely to significantly change the relations that are already embedded in the network. For this reason high disease correlations seem to be more informative. In other words, a high correlation between two diseases is suggestive of a relationship between the two, but a low correlation may just reflect that there is not enough information in the network. Even for very highly correlated (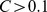) diseases, our approach still could not find common pathways for all disease pairs. This could still be due to incompleteness of the network or because of the fact that our method uses a rather simple measure to investigate possible disease relations.

In summary, we have proposed to use network-based correlations between diseases as a measure of diseases similarity. Higher correlations could be interpreted as a higher probability for the disease pairs to have common biological pathways/processes. Despite its simplicity and limitations, the simple approach employed seems to be able to, in most cases, distinguish between disease pairs with and without shared GO/KEGG biological terms as well as properly group diseases sharing similar biological processes/pathways.

## Supporting Information

Supporting Information S1 All supporting information are given in this file, including a description of how cutoffs were calculated for MimMiner score and correlation, the results of the evaluation of the accuracy of the p-values, and also the results of clustering using Cfinder. **Figure S1**, Finding the optimum number of clusters. Figure shows (A) the number of clusters, and (B) R, as a function of number of iterations. R is minimized after 10 iterations. **Figure S2**, Empirical p-values vs p-value cutoffs. The empirical values were calculated by shuffling the gene list 672 times. **Figure S3**, The probability of finding shared KEGG pathways is plotted (in red) as a function of average MimMiner score (a) or average correlation (b). The blue line shows the fitted piecewise function. The separation points are considered the cutoffs above which the scores or correlations are significant.
(PDF)
