# Supplementary material for: Relating Diseases by Integrating Gene Associations and Information Flow through Protein Interaction Network
Source: PLoS One. 2014 Oct 31;9(10):e110936. doi: 10.1371/journal.pone.0110936 (PMC4216010; doi:10.1371/journal.pone.0110936)
Supplement: Supporting Information S1 — All supporting information are given in this file, including a description of how cutoffs were calculated for MimMiner score and correlation, the results of the evaluation of the accuracy of the p-values, and also the results of clustering using Cfinder. Figure S1, Finding the optimum number of clusters. Figure shows (A) the number of clusters, and (B) R, as a function of number of iterations. R is minimized after 10 iterations. Figure S2, Empirical p-values vs p-value cutoffs. The empirical values were calculated by shuffling the gene list 672 times. Figure S3, The probability of finding shared KEGG pathways is plotted (in red) as a function of average MimMiner score (a) or average correlation (b). The blue line shows the fitted piecewise function. The separation points are considered the cutoffs above which the scores or correlations are significant. (PDF) [file pone.0110936.s001.pdf]

Supporting information for the manuscript "Relating diseases by integrating gene associations and information flow through protein interaction network"

Mehdi Bagheri Hamaneh and Yi-Kuo Yu

Clustering

Figure S1 shows the number of clusters and the quantity R as a function of number of steps in the iterative clustering method descrined in the Methods.

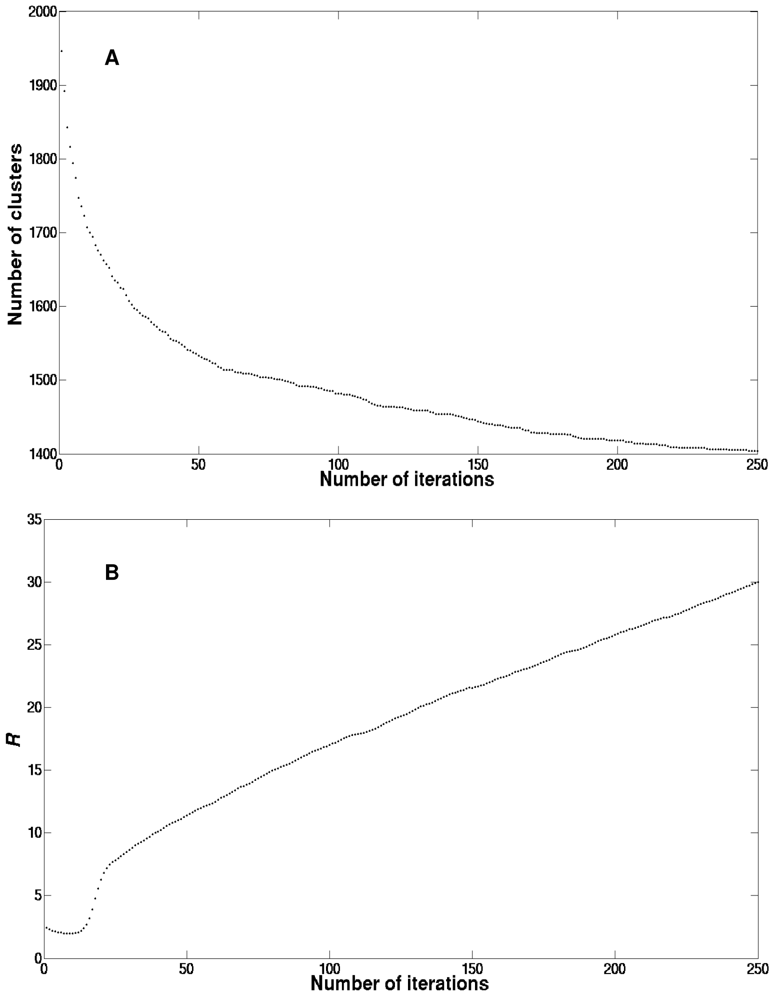

Figure S1: **Finding the optimum number of clusters.** Figure shows (A) the number of clusters, and (B) R, as a function of number of iterations. R is minimized after 10 iterations.

Clustering was also performed using Cfinder, a program developed for overlapped clustering of networks [?, ?]. The program finds the union of all adjacent complete subgraphs of size $k \geq 3$ (subgraphs consisting of k nodes that are all linked to each other) that can be reached from each other. Two such subgraphs, or k-cliques, are said to be adjacent if they have $k-1$ common nodes. The intensity of a

k-clique is defined as the geometric average of the weights of its links. For clustering weighted networks, Cfinder requires two parameters to be set: 1) k which determines the size of the k-cliques considered, and 2) the intensity threshold, denoted by I. The user has also the option to specify a cutoff (W) to speed up the program. If specified, all links with weights smaller than W are ignored. In this study we chose $W = \langle C \rangle = 5.4 \times 10^{-4}$, because Cfinder was very slow for $W \sim 10^{-4}$ and smaller cutoffs. We followed the recommended procedure in [?] to determine the optimal value of I i.e. we ran Cfinder for different values of k (ranging from 3 to 6), and for each k, we found the lowest $I(k)$ for which the size of the largest cluster was less than two times that of the second largest cluster. To find the best value for k, the quantity $\tilde{R}(I(k), k) = n_c \langle N \rangle$ was calculated, where n_c is the number of clusters and $\langle N \rangle$ denotes the average number of clusters that each disease is a member of. Conceptually, $\tilde{R}$ is very similar to R defined in the Methods. On the one hand, it is desirable to have the lowest number of clusters possible. On the other hand, the diseases should not be in too many clusters. Therefore, we chose a value for k that minimized $\tilde{R}(I_m(k), k)$, where $I_m(k)$ is the optimal I corresponding to k.

The optimal values of k and I found to be 3 and 0.11 respectively. Out of 2534 diseases, 1155 were clustered into 298 clusters. Considering the rest of the diseases as clusters with only one member, the total number of clusters was 1677 (close to the number of clusters obtained from the clustering method presented in this paper, i.e. 1707). To compare the results of Cfinder to those of our probabilistic clustering approach, for each disease pair we calculated $\tilde{\rho}_{ij} = \frac{N_{ij}}{\sqrt{N_i N_j}}$, where N_{ij} is the number of clusters that contain both disease i and disease j and N_i is the number of clusters that include disease i. $\tilde{\rho}_{ij}$ is a measure of similarity of the memberships of the two diseases, i.e. if diseases i and j belong to totally different sets of clusters, $\tilde{\rho}_{ij}$ will vanish, and if the two diseases are in the same clusters it will be equal to unity. The corresponding quantity in our clustering algorithm can be defined as $\rho_{ij} = \cos(\mathbf{p}_i, \mathbf{p}_j)$, where $\mathbf{p}_i$ is a vector whose elements are the membership probabilities of disease i. The disease pairs were divided into two groups: Group 1 consisted of pairs of different diseases with $\tilde{\rho}_{ij} > 0$ (the two diseases shared at least one cluster), and Group 2 included disease pairs with $\tilde{\rho}_{ij} = 0$, i.e with no shared clusters. Interestingly, $\langle \tilde{\rho}_{ij}^{(1)} \rangle$ and $\langle \rho_{ij}^{(1)} \rangle$ were determined to be 0.68 and 0.66 respectively, where the superscript indicates that averaging was performed over Group 1. The corresponding standard deviations were 0.27 and 0.25. Considering $\rho_{ij}^{(1)}$ and $\tilde{\rho}_{ij}^{(1)}$ as one-dimensional vectors, we also calculated the cosine of the angle between these two vectors and found it to be 0.94. On the other hand, $\langle \rho_{ij}^{(2)} \rangle$ was 0.02 (with a standard deviation of 0.05). These statistics suggest that the two clustering methods result in similar disease-cluster memberships.

For each cluster (with more than one member) found by Cfinder, a cluster center was defined as the mean of the weight vectors of the diseases in that cluster. Enrichment analysis was then performed for each cluster center (using the same parameters mentioned in the Methods). For each disease, the associated biological terms were then determined as the union of the terms associated with all clusters that the disease was a member of. As expected, this procedure increased the percentage of diseases with term hits from 60% to 0.66%. For the 1155 diseases that had been clustered by Cfinder this percentage was increased from 73% to 86%. As specific examples, once again we consider Parkinson's disease, Retinitis Pigmentosa type 7, and Knobloch syndrome. Knobloch syndrome, which was primarily in one cluster using our clustering method, was not clustered by Cfinder, and so was not associated with any terms. Retinitis Pigmentosa type 7, on the other hand, was a member of one cluster that also included Vitelliform macular dystrophy (OMIM:608161), Fundus albipunctatus (MEDH:C562733) and Central areolar chorodial dystrophy 2 (MESH:C567750), which is a subset of the cluster shown in Fig. 5. The terms associated with this cluster were very similar to the ones reported in Table 3 including GO:0007603 (phototransduction of visible light), GO:0009584 (detection of visible light) and GO:0009583 (detection of light stimulus). Two clusters included Parkinson's disease as a member: one containing (in addition to Parkinson's disease) MESH:C535600 (Dopamine beta hydroxylase defiency) and MESH:D010259 (Paranoid disorders), and the other with OMIM:601696 and MESH:D001010. Both clusters were associated with GO:0019226 (transmission of nerve impulse), GO:0007268 (synaptic transmission), GO:0035637 (multicellar organismal signaling), GO:0007267 (cell-cell signaling), and GO:0050877 (neurological system process), which are among the terms associated with this disease using the clustering approach proposed here. However,

only one of the clusters was associated with KEGG:hsa05012 (Parkinson's disease). Overall, the data indicates that Cfinder clustering associates Parkinson's diseases to very similar set of terms as does the probabilistic clustering approach.

Accuracy of the p-values

To investigate how clustering (averaging the weights) might affect the accuracy of the reported p-values, and therefore the E-values, enrichment analyses were run for randomly shuffled gene lists and empirical p-values were calculated (see Methods). Figure S2 shows that empirical p-values are consistently lower than the p-value cutoffs. This is in agreement with what was found [**?**] for original (not averaged weights), and indicates that the reported p-values are even more conservative than the empirical ones.

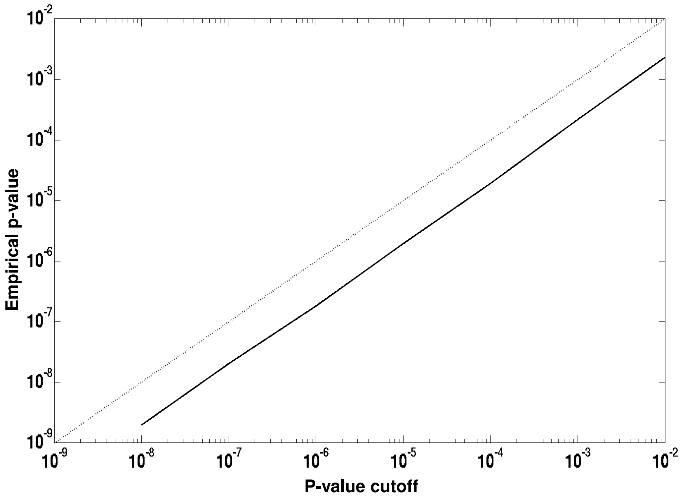

Figure S2: Empirical p-values vs p-value cutoffs. The empirical values were calculated by shuffling the gene list 672 times.

Cutoffs for MimMiner score and correlation

The correlation defined in this paper and the score provided by MimMiner have very different scales, and are defined based on different concepts. Therefore, it is not obvious how one can find the corresponding MimMiner score for a given correlation. On the other hand, it is desirable to determine cutoffs for both measures above which disease-disease similarities can be considered significant. These cutoffs, which are considered equivalent, could also relate the two measures (although only at one point).

To estimate the cutoffs, we used KEGG disease-pathway associations as a "gold standard". All disease pairs with defined KEGG term similarities (S) were ranked based on either their correlations or their scores, creating two lists of pairs. The two lists had 808356 (our method) and 669903 (MimMiner) members. The pairs in each list were binned (1000 pairs per bin) and for pairs in each bin the probability of having a common KEGG pathway ($S_{\rm bav}$) was calulated. The results, shown in Figs. S3 (a) and (b), demonstrate a similar trend for both cases, i.e. the probability remains low and flat when correlation/score is low and starts almost a linear increase when disease similarity becomes larger than a threshold. It should be noted that the scale of average correlation in Fig. S3 (b) is logarithmic. In each case, to find the point that the probability starts to deviate from the flat background (the cutoff), a piecewise function was fitted to the data (the blue line in the figure). The function was assumed to be constant at low similarities, but rising linearly (in the case of MimMiner Fig. S3 (a)) or proportional to $\log(C_{\rm bav}) - \log(C_{\rm cutoff})$ (in

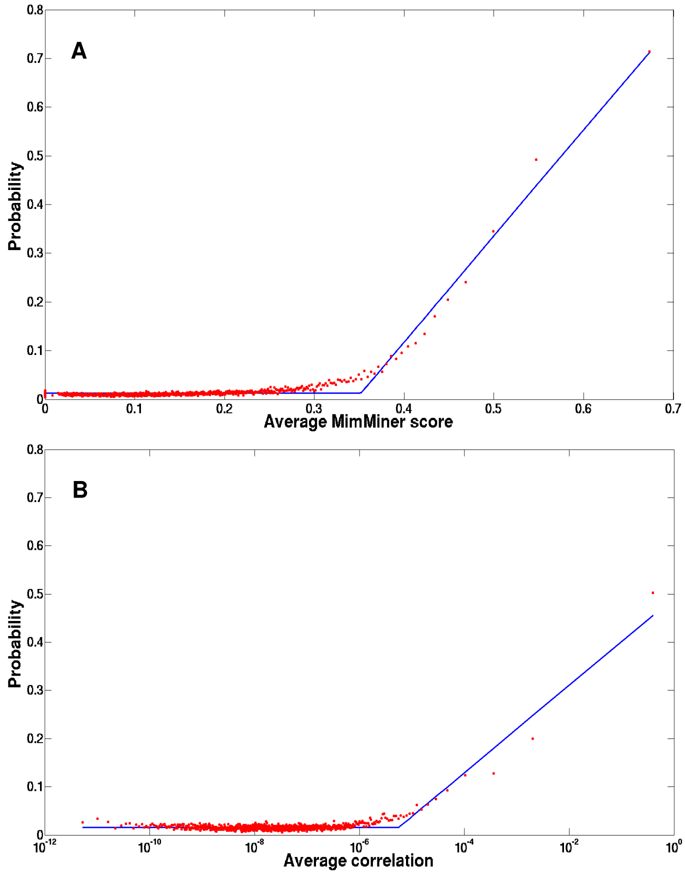

Figure S3: The probability of finding shared KEGG pathways is plotted (in red) as a function of average MimMiner score (a) or average correlation (b). The blue line shows the fitted piecewise function. The separation points are considered the cutoffs above which the scores or correlations are significant.

the case of our method Fig. S3 (b)) after a cutoff. The cutoffs, calculated by least square fitting, were found to be $5.7e-6$ and 0.35 for correlation and MimMiner score respectively.
